# Supplementary material for: Identification of Freezing-Responsive microRNAs and Their Targets in Chinese Jujube by Small RNA and Degradome Sequencing
Source: Int J Mol Sci. 2026 Sep 4;27(17):7912. doi: 10.3390/ijms27177912 (PMC13566890; doi:10.3390/ijms27177912)
Supplement: Supplementary file 1 [file ijms-27-07912-s001.zip › Supplementary Material S2/novel_miR_72.pdf]

Provisional ID	:	novel_miR_72
Score total	:	0.9
Score for star read(s)	:	-1.3
Score for read counts	:	0
Score for mfe	:	0.6
Score for randfold	:	1.6
Score for cons. seed	:	
Total read count	:	259
Mature read count	:	257
Loop read count	:	2
Star read count	:	0

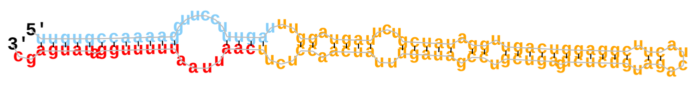

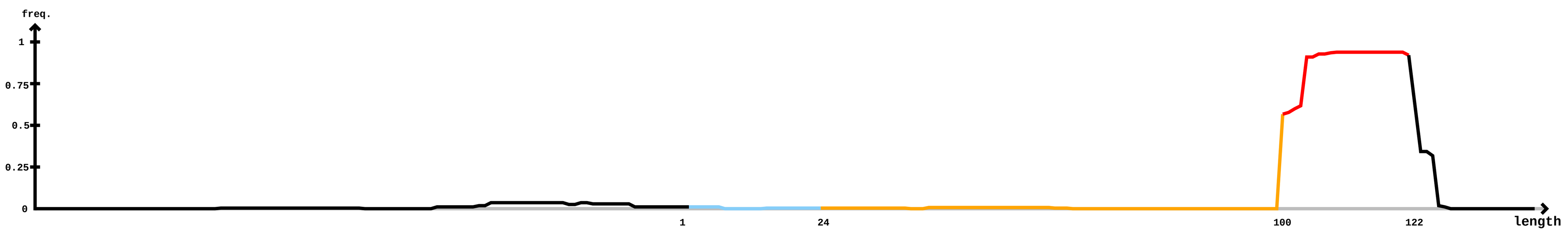

Star
Mature

```
5'- ugaaauuuugaaaguaauuuuguacugucaaagauucuuucuguugauuaugauuuaguuagauccuaaaucauauggaucuugacuuucuugauagucggcccaucuugugccaaaagguuccuuugauuuggaugauucugcuauagguugacuggaggcuucaucagaugucucugagucguccgauaguuuaucaaccucuucaauuaauuuuuggauaugagcaagcaucuccuucuuugaucug -3'   exp
    .(((.((((((.((((......)))).)))))).))).....(((((((((.(...((((((((((..........))))).)))))....).)))))))))....(((((((((((((......((((...((.((((...(((((.((.(((((((((((.((....)).)))))).))))).)).)))))..)))).))...))))....))))))).))))))..((.(((.........))))).   reads   mm   sample
    .............................aaagauucCuucuguugauuauga.....................................................................................................................................................................................................   1   1   JCK-3
    .................................................................cuaaaucauaGgaucuugacu....................................................................................................................................................................   3   1   LT-2
    ........................................................................auauggaucuugacuuucu...............................................................................................................................................................   2   0   LT-1
    ..........................................................................auggaucuugacuGucuugauagu........................................................................................................................................................   3   1   JCK-2
    ..........................................................................auggaucuugacuGucuugauagu........................................................................................................................................................   2   1   JCK-1
    .........................................................................................cuugauagucggcccaucuugugc.........................................................................................................................................   3   0   LT-2
    ........................................................................................................................uuccuuugauuuggaugauucugc..........................................................................................................   1   0   LT-1
    ...................................................................................................................................................agguugacuggaggcuucauc..................................................................................   1   0   LT-1
    ...................................................................................................................................................agguugacuggaggcuucaucaga...............................................................................   1   0   LT-3
    ..............................................................................................................................................................................................................caauuaauuuuuggauaugagc......................   2   0   LT-1
    ..............................................................................................................................................................................................................caauuaauuuuuggauaugagc......................   16   0   LCK-2
    ..............................................................................................................................................................................................................caauuaauuuuuggauaugagc......................   30   0   JCK-3
    ..............................................................................................................................................................................................................caauuaauuuuuggauaugagc......................   47   0   JCK-2
    ..............................................................................................................................................................................................................caauuaauuuuuggauaugagc......................   17   0   JCK-1
    ..............................................................................................................................................................................................................caauuaaCuuuuggauaugagc......................   1   1   LT-3
    ..............................................................................................................................................................................................................caauuaauuuuuggauaugagc......................   3   0   LCK-3
    ..............................................................................................................................................................................................................caauuaauuuuuggauaugagc......................   16   0   LCK-1
    ..............................................................................................................................................................................................................caauuaauuuuuggauaugagc......................   10   0   LT-2
    ..............................................................................................................................................................................................................caauuaauuuuuggauaugagc......................   9   0   LT-3
    ..............................................................................................................................................................................................................caauuaauuuuuggauaugagca.....................   1   0   LT-1
    ..............................................................................................................................................................................................................caauuaauuuuuggauaugagca.....................   3   0   JCK-2
    ..............................................................................................................................................................................................................caauuaauuuuuggauaugagca.....................   2   0   LCK-3
    ...............................................................................................................................................................................................................aauuaauuuuuggauaugagc......................   2   0   JCK-1
    ...............................................................................................................................................................................................................aauuaauuuuuggauaugagc......................   1   0   JCK-3
    ................................................................................................................................................................................................................auuaauuuuuggauaugag.......................   1   0   JCK-1
    ................................................................................................................................................................................................................auuaauuuuuggauaugag.......................   4   0   JCK-2
    ................................................................................................................................................................................................................auuaauuuuuggauaugagcaagc..................   1   0   LT-3
    .................................................................................................................................................................................................................uuaauuuuuggauaugagcaag...................   2   0   JCK-2
    .................................................................................................................................................................................................................uuaauuuuuggauaugagcaag...................   2   0   JCK-3
    .................................................................................................................................................................................................................uuaauuuuuggauaugagcaag...................   1   0   JCK-1
    ..................................................................................................................................................................................................................uaauuuuuggauaugagcaag...................   1   0   JCK-2
    ..................................................................................................................................................................................................................uaauuuuuggauaugagcaag...................   1   0   JCK-3
    ..................................................................................................................................................................................................................uaauuuuuggauaugagcaagc..................   14   0   JCK-2
    ..................................................................................................................................................................................................................uaauuuuuggauaugagcaagc..................   8   0   JCK-3
```

Star Mature

ugaaauuuugaaaguaauuuuguacugucaaagauucuuucuguugauuaugauuuaguuagauccuaaaucauauggaucuugacuuucuugauagucggcccaucuugugccaaaagguuccuuugauuuggaugauucugcuauagguugacuggaggcuucaucagaugucucugagucguccgauaguuuaucaaccucuucaauuaauuuuuggauaugagcaagcaucuccuucuuugaucug			
....uaauuuuuggauaugagcaagc..................	11	0	JCK-1
....uaauuuuuggauaugagcaagc..................	1	0	LT-3
....uaauuuuuggauaugagcaagc..................	18	0	LCK-1
....uaauuuuuggauaugagcaagc..................	25	0	LCK-2
....uaauuuuuggauaugagcaagc..................	2	0	LT-1
....auuuuuggauaugagcaagc..................	1	0	JCK-1
....auuuuuggauaugagcaagc..................	2	0	LT-3
....auuuuuggauaugagcaagcau................	1	0	JCK-3
....auuuuuggauaugagcaagcau................	1	0	JCK-1
....uuuuggauaugagcaagca.................	2	0	LT-3
....uuuggauaugagcaagcau................	1	0	LT-3
